# *De novo* transcriptome sequencing and assembly from apomictic and sexual *Eragrostis curvula* genotypes

**DOI:** 10.1371/journal.pone.0185595

**Published:** 2017-11-01

**Authors:** Ingrid Garbus, José Rodolfo Romero, Juan Pablo Selva, María Cielo Pasten, Carolina Chinestra, José Carballo, Diego Carlos Zappacosta, Viviana Echenique

**Affiliations:** 1 Centro de Recursos Naturales Renovables de la Zona Semiárida (CERZOS) Universidad Nacional del Sur-CONICET, Bahía Blanca, Argentina; 2 Departamento de Ciencias de la Salud, Universidad Nacional del Sur (UNS), Bahía Blanca, Argentina; 3 Departamento de Biología, Bioquímica y Farmacia, Universidad Nacional del Sur (UNS), Bahía Blanca, Argentina; 4 Departamento de Agronomía, Universidad Nacional del Sur (UNS), Bahía Blanca, Argentina; Georg-August-Universitat Gottingen, GERMANY

## Abstract

A long-standing goal in plant breeding has been the ability to confer apomixis to agriculturally relevant species, which would require a deeper comprehension of the molecular basis of apomictic regulatory mechanisms. *Eragrostis curvula* (Schrad.) Nees is a perennial grass that includes both sexual and apomictic cytotypes. The availability of a reference transcriptome for this species would constitute a very important tool toward the identification of genes controlling key steps of the apomictic pathway. Here, we used Roche/454 sequencing technologies to generate reads from inflorescences of *E*. *curvula* apomictic and sexual genotypes that were *de novo* assembled into a reference transcriptome. Near 90% of the 49568 assembled isotigs showed sequence similarity to sequences deposited in the public databases. A gene ontology analysis categorized 27448 isotigs into at least one of the three main GO categories. We identified 11475 SSRs, and several of them were assayed in *E curvula* germoplasm using SSR-based primers, providing a valuable set of molecular markers that could allow direct allele selection. The differential contribution to each library of the spliced forms of several transcripts revealed the existence of several isotigs produced via alternative splicing of single genes. The reference transcriptome presented and validated in this work will be useful for the identification of a wide range of gene(s) related to agronomic traits of *E*. *curvula*, including those controlling key steps of the apomictic pathway in this species, allowing the extrapolation of the findings to other plant species.

## Introduction

Apomixis, a suite of mechanisms leading to asexual seed production, is an example of a complex trait for which comparative transcriptomics is a useful approach. In contrast to sexual seed formation, apomictic reproduction avoids meiosis and fertilization to generate an embryo that is solely maternal in genotype. The maternal genotype remains fixed and is stably maintained, barring mutation, from one seed generation to the next [1]. Despite the importance of fixing heterosis in hybrid species, understanding the nature of apomixis remains a challenge.

Expression-based strategies have been used with apomictic species, under the rationale that spatial and/or temporal changes in the abundance of particular gene transcripts between apomicts and sexual genotypes would reveal the differential transcriptional pathways involved in apomixis [2, 3].

Carman [4] proposed that apomixis results from the temporal deregulation of cell specification and fate during early ovule development, taking advantage of polyploidy either by alleviating self-incompatibility [5], or by masking the effects of deleterious mutations [6]. For instance, in *Boechera* species, apomeiosis was correlated with the global downregulation of gene expression during megaspore mother cell formation [7]. Though it is well established that apomixis is inherited as a dominant trait, and dominant loci that independently control diplospory/apospory and parthenogenesis have been identified in several species [6, 8–12], the genetic basis of its regulation remains unclear [13, 14]. Moreover, additional effectors such as ploidy [6, 15, 16] and environmental factors [17, 18] disguise the genetic basis of this trait.

Apomixis loci in different species have also been associated with heterochromatin and/or repetitive sequences that supposedly have a functional role in apomixis [19, 20]. However, recent results suggest that these structural features and allelic divergence are a consequence of asexual reproduction and suppressed recombination, which may have evolved to maintain genic elements required for apomixis [21].

*E*. *curvula* (Schrad.) Nees (weeping lovegrass) is a perennial grass member of the Poaceae family, subfamily Chloridoideae [22]. It is native to southern Africa and cultivated in semiarid regions [23]. The *E*. *curvula* complex has a basic chromosome number of × = 10 [24] and includes cytotypes with different ploidy levels (from 2–8X) that may undergo sexual reproduction, facultative or obligate apomixis [25]. Diploid (2*n* = 2X = 20) plants are sexual, rare, and do not occur in all forms of *E*. *curvula* [26]. Tetraploid plants (2*n* = 4X = 40) and plants with high ploidy levels reproduce by pseudogamous diplosporous apomixis [25, 27, 28]. The type of embryo sac development in *E*. *curvula* is particular of this grass (*Eragrostis*-type) and contains only four non-reduced nucleus at maturity [27]. Meiotic divisions are absent from the *Eragrostis*-type apomixis, and the megasporocyte undergoes only two rounds of mitotic division to form a non-reduced tetranucleated embryo sac with an egg cell, two synergids, and one polar nucleus [29]. Embryos are formed by parthenogenesis, and fertilization of the polar nuclei (pseudogamy) is necessary for endosperm development.

By constructing expressed sequence tag (EST) libraries, our group has previously conducted transcriptome studies to compare near-isogenic diplosporous and sexual *E*. *curvula* genotypes [30]. Analysis of these libraries, together with differential display studies conducted with these genotypes, allowed the identification of a set of genes differentially expressed between diplosporous and sexual *E*. *curvula*, including some coding for retrotransposon proteins [30–33]. However, the number of genes sequenced with these technologies was quite low compared to the number of genes that the Next-generation sequencing (NGS) technologies could provide. These technologies are revolutionizing our ability to characterize traits or diseases at the genomic, transcriptomic and epigenetic levels. NGS have been particularly useful in constructing libraries to transcripts characterization, including comparisons of gene expression between different genotypes, developmental stages and tissues.

In this work a reference floral transcriptome of *E*. *curvula* was obtained using the Roche 454 NGS technology aiming to increase the number of available *E*. *curvula* unigenes. To carry out our purpose, four cDNA libraries were constructed from sexual and full apomictic-derived inflorescences. We hypothesized that the reference transcriptome for this species would constitute a key step for the identification of the gene or genes involved in triggering or controlling the apomictic pathway. This, in turn, might reveal the precise mechanisms of apomixis in this species, and allow us to extrapolate our findings to other plant species.

## Materials and methods

### Plant materials

Two tetraploid accessions of *Eragrostis curvula* (weeping lovegrass) provided by the United States Department of Agriculture (USDA) were used in this work: the full apomictic genotype ‘Tanganyika’ (USDA: PI234217) (Ec-T, 2n = 4× = 40), and the sexual genotype ‘OTA-S’ (USDA: PI574506) (Ec-S, 2n = 4× = 40). For SSR validation, other genotypes with different ploidy levels and/or reproductive modes were used (Table 1). Plants were grown in a greenhouse, under natural light conditions at 25°C.

**Table 1:** Genotypes assayed to simple sequence repeat (SSR) validation.

Abbreviated name	Genotype	Ploidy	Reproductive mode
1	PI299920	2X	Sexual
2	PI208214	2X	Sexual
3	PI299919	2X	Sexual
4	PI219928	2X	Sexual
5	OTA-S	4X	Sexual
6	Victoria	2X	Sexual
7	Tanganyika USDA	4X	Apomictic
8	Don Walter	4X	Apomictic
9	Ermelo	4X	Apomictic
10	Morpa	4X	Apomictic
11	Don Pablo	7X	Apomictic
12	Tanganyika INTA	4X	Apomictic
12	UNST9355	6X	Apomictic
14	Don Luis	6X	Apomictic

### RNA isolation and preparation

Spikelets with basal flowers at the beginning of anthesis, containing embryo sacs at all developmental stages, were collected from OTA-S and Tanganyika plants. Four RNA samples were prepared: O2P1 and O2P2 were biological replicates derived from spikelets of sexual genotype OTA-S whereas T3P1 and T3P2 were biological replicates obtained from spikelets of the full apomictic genotype Tanganyika. For each sample, 30 mg of fresh tissue was ground to a fine powder in liquid nitrogen, and the total RNA was extracted from plant tissue as two fractions, small and large RNA, including RNA sequences smaller and larger than 200 bp, respectively, using a commercial RNA purification kit (Macherey-Nagel) according to the manufacturer’s instructions.

The large RNA fraction was resuspended in 100 μl diethylpyrocarbonate (DEPC)-treated water and quantified using the cyanine dye RiboGreen^®^ (Molecular Probes, Eugene, OR, USA) [34], against an appropriate standard curve, as described by the manufacturer. Quantities of RNA ranged between 37 to 60 μg. RNA quality was checked with a RNA 6000 Pico LabChip using the Agilent 2100 Bioanalyzer instrument (Agilent, USA). mRNA was purified using Dynabeads oligo (dT) magnetic beads (Invitrogen), following the manufacturer protocol, and quantified using an Agilent 2100 Bioanalyzer instrument (Agilent, USA). A total of 200 ng of each RNA sample meeting the quality criterion of integrity was used for chemical fragmentation of mRNA, as recommended by the cDNA Rapid Library Preparation Method Manual (Roche). Fragmentation of the samples was checked by comparison with non-fragmented samples after running on the RNA 6000 Pico Chip on the Agilent 2100 Bioanalyzer. The cDNA library was constructed using the GS FLX Titanium Rapid Library Preparation Kit (Roche). Briefly, double-stranded cDNA synthesis was conducted using the cDNA Synthesis System Kit (Roche), followed by a double-stranded cDNA purification and a fragment end repair step. Then, adaptors were ligated, according to RL FLX+ (Roche) cDNA Rapid Library Preparation Method Manual (May 2011). Library quality assessment was carried out using the Agilent Bioanalyzer High Sensitivity DNA chip (Agilent). Samples were titrated by conducting emulsion PCR at large scale (LV-emPCR) following the GS Titanium LVemPCR Kit Lib-L v2 (Roche) manual, under the criteria that enrichments of 5–20% would retrieve adequate sequencing results. Sequencing was carried out using the Roche 454 GS FLX pyrosequencing platform at INDEAR (Argentina), using half of a titanium plate for each library, according to the protocol described by the manufacturer.

### De novo assembly

The quality control of the resulting raw sequence reads was evaluated through PRINSEQ [35], which generated summary statistics of the input sequence files and quality data, including length distribution, GC content distribution, base quality distribution, and occurrence of N, Poly-A/T Tails, tag sequence check, sequence duplication and sequence complexity. PRINSEQ was also used for filtering and trimming of the data. Sequences with a mean quality score lower than 20 and more than 5% of occurrence of N were filtered out. The trimming of sequences was performed before filtering steps by removing the adaptors from the 5′- and 3′-ends. Then, curated raw read sequences were assembled into contigs, isotigs, and isogroups using Newbler Assembler software version 2.6 (Roche, IN, USA). The only deviation from default settings was the application of the [–urt] option to improve the production of contigs in low depth regions of the assembly.

### Assembly validation

To validate the transcript assembly, whole assembled transcripts were initially aligned using a BLAST algorithm [36] run through NCBI-BLAST-2.4.0+ (version 2.4) against: a) a local database previously constructed from *E*. *curvula* ESTs EH183417 to EH195711 (BLASTN; e-values: e^-10^ and e^-100^); b) 4541 cDNA *E*. *tef* sequences from NCBI ESTs database (BLASTN; e value < e^-10^; coverage > = 40%). The same algorithm was then applied to compare *E*. *curvula* with *E*. *tef* whole genome shotgun (WGS) sequences LAPY01000001 to LAPY01013883 [37]; (BLASTN; e-value: e^-10^; isotig coverage > = 10%). Then, 454 reads were mapped to *E*. *tef* whole genome shotgun (WGS) sequences LAPY01000001 to LAPY01013883 [37], using the software GMAP [38] (coverage > 80%; identity >85%).

A subgroup of sequences was also searched for chimeric assemblies. This subgroup comprised sequences of 3850–15244 bp in size, which included only one sequence per isogroup. Sequences matching thBe NCBI protein (nr) database (https://www.ncbi.nlm.nih.gov/protein) with a coverage ≥ 85% were not considered chimeric, while those with < 85% coverage were manually analyzed.

### Functional annotation

BLASTX (e-value cut off ≤ 10e^-05^) searches were first performed against the nonredundant protein database at NCBI (nr, version 2.2.29). Sequences without hits were used to perform successive BLASTX searches against the PROSITE (http://prosite.expasy.org) and dbEST (https://www.ncbi.nlm.nih.gov/dbEST/) databases to assess their putative identities and/or predicted functions. BLAST2GO platform [39] was used to assign gene ontology annotation using a combination of similarity searches and statistical analysis.

### Plastidic unigene identification

To search for mitochondrial and chloroplast sequences, the assembled reads (isotigs) were aligned using a BLAST algorithm run through NCBI-BLAST-2.4.0+ (version 2.4) against local databases constructed from 406 mitochondrial and 101 chloroplast genome sequences, and 4814 mitochondrial and 5810 chloroplast proteins, downloaded from the NCBI nucleotide collection (https://www.ncbi.nlm.nih.gov/nuccore) and Chloroplast DB (http://chloroplast.cbio.psu.edu/index.html).

### Differential expression analysis

Prior to differential gene expression analysis, read counts for each sequenced library were adjusted by one scaling normalized factor using the edgeR software package [40]. This function normalizes for RNA composition by finding a set of scaling factors for the library sizes that minimize the log-fold changes between samples for most genes. No filtering was applied before the analysis. The default method for computing these scale factors uses a trimmed mean of M-values (TMM) between each pair of samples [41]. The product of the original library size and the scaling factor is called the effective library size—this replaces the original library size in all downstream analyses. The dispersion parameter for each tag, measure of the degree of inter-library variation for that tag, was estimated using empirical Bayes tagwise dispersions. The square root of the common dispersion gives the coefficient of variation of biological variation (BCV). Once the dispersions were estimated, testing procedures for determining differential expression were conducted. An exact test conducts tagwise tests using the exact negative binomial test proposed by Robinson and Smyth [42]. Differential expression between two conditions was analyzed using the EdgeR package [40], and P-values adjusted using the Benjamini and Hochberg method [43], to control the false discovery rate. The corrected P-value of 0.005 and log2 (fold-change) of 1 were set as the threshold for significant differential expression. A plot of the tagwise log-fold-changes against log-count-per-million was generated, highlighting the DE genes. This pipeline is primarily intended for use with data that include biological and technical replicates; however, because no technical replicates were used in this study, a reasonable dispersion value of bcv = 0.01 was chosen for the Exact Test calculation.

For validation assays, total RNA was isolated from inflorescences from Tanganyika and OTA-S, following the protocol previously used for library construction. Reverse transcription was performed as described in [33]. Amplification of the cDNA was performed using specific primer pairs designed and synthesized through the Integrated DNA technology (IDT) webpage (http://www.idtdna.com/Scitools/Applications/RealTimePCR/). The RT-PCR program consisted of a denaturation step at 94°C for 2 min followed by 40 cycles at 94°C for 15 s, 20 s at the optimal annealing temperature for each primer pair, and at 72°C for 30 s, followed by an extension step at 72°C for 5 min. Real time PCR reactions, included 50 pmol of forward and reverse primers, 5 μl of cDNA diluted 100-fold, and 10 μl of Real Mix (Biodynamics, Buenos Aires, Argentina). Amplification was carried out in a Rotor Gene 6000 (Corbett Research, Sydney, Australia). Cycling consisted of 94°C for 2 min followed by 45 cycles at 94°C for 15 s, for 20 s at the optimal annealing temperature for each primer pair, and at 72°C for 30 s. After that, to control the specificity of amplification products, a melting curve was run, consisting of cycles of 10 s from 72 to 95°C, increasing the temperature by 0.5°C in each cycle after cycle 2. The reactions were conducted in biological duplicates. The ubiquitin conjugating enzyme transcript, used as housekeeping gene, was amplified with *E*. *curvula*-based primers (UBICE_F / R: AAGGAGCTCAAGGACCTGCAGAAA / TCACTAAGAACACACCACCGGCAT) [33]. The isotig-based primers designed for validation were isotig27875_F / R: GCTGGAGTCTTAGGGGCTTT / AGACACCTACAGACGGCAAA; isotig38926_F / R: TGCACTGTTGAGGATGAAGC / CTTGTGGAGAGGTATGGGGA and isotig36655_F / R: AAAGGCCTGAATGCTCTGAA / TTCAGCTCGGGACTTCATCT.

Isotigs differentially represented between sexual and apomictic libraries were subjected to GO analysis. Distribution of the GO annotation frequency and representation in both reproductive modes was statically evaluated by analysis of variance (ANOVA) and regression analysis via Infostat (http://www.infostat.com.ar) where ANOVA indicated significant differences between samples, and mean separation was determined using Fisher’s least significant difference (LSD) test at *P* = 0.05.

### Development and detection of gene markers: Transcriptome-derived simple sequence repeats (SSRs)

Assembled isotigs were used as the input data sequence to develop simple sequence repeats (SSRs) using SSR locator software [44]. A minimum of ten repeats was considered for mononucleotide, six for dinucleotide, and 5 for trinucleotide, tetranucleotide, pentanucleotide and hexanucleotide motifs. The minimum distance between two different SSRs was set to 100 bp. Primers flanking each SSR were designed using the software Primer3 (2.3.5 version) with default parameters (http://bioinfo.ut.ee/primer3-0.4.0/). Primer design criteria were selected as follows to obtain amplification products of 150–250 bp: annealing temperature 50–65°C, with an optimum value of 55°C; CG content of 20–50%; 15–25 bp (optimum 20 bp), end stability 250 kcal/mol. Sequence specificity was checked using the software PrimerSearch (http://www.bioinformatics.nl/cgi-bin/emboss/primersearch) against the assembled isotigs (mismatch = 0), thus primers were synthesized to be specific to a single isotig, or—eventually—to a group of isotigs belonging to the same isogroup.

Thirty five primer pairs were synthesized (Genbiotech, Buenos Aires, Argentina) and assayed in polymerase chain reaction (PCR) amplification reactions with 14 different genotypes of *E*. *curvula*. Genomic DNA was extracted from fresh leaf tissue following a protocol based on cetyltrimethylammonium bromide (CTAB). Briefly, plant material was frozen and powdered in liquid nitrogen by using TissueLyser II (Qiagen). Then, 100mg of powder was incubated at 65°C in preheated extraction buffer containing 100 mM Tris HCl pH 8, 1.4 M NaCl, 20 mM EDTA pH 8, 2% CTAB (w/v) and 0.5% (v/v) β-mercaptoethanol. Then, chloroform was added to reach the proportion 2:1, and supernatant obtained after centrifugation was collected. Finally, DNA was precipitated with one volume of isopropanol, washed with 70% (v/v) ethanol and resuspended in TE buffer containing 20ug/ml of RNase. PCR amplification was conducted using a MyCycler Biorad cycler. Each PCR reaction used 1 μl of 10 mM dNTPs mix, 2.5 μl of 10× reaction buffer, 0.5 μl of each forward and reverse primer (100 pmol/μl), 0.30 μl of DNA polymerase Taq Pegasus (5 U/μl) and 2 μl of template genomic DNA (30 ng/μl), in a final reaction volume of 25 μl.

The PCR reaction profile was: initial DNA denaturation at 94°C for 3 min, followed by 40 cycles at 94°C for 30 s, 30 s at the optimal annealing temperature for each primer pair, and 72°C for 30 s. The third step consisted of a final extension of 5 min at 72°C. The annealing temperatures for each primer pair were set as the lower melting temperature of both primers. Primers that did not amplify under these initial conditions were subjected to further amplifications decreasing by 1°C decrease each time, while for primers producing multiple bands, the annealing temperature was adjusted by 1°C increases.

Amplicons were analyzed by electrophoresis in 2% (m/v) agarose gel and 6% polyacrylamide gel after mixing the samples with loading buffer. For electrophoresis in agarose gels, the fragments were visualized using ethidium bromide, while those run in acrylamide gels were visualized by silver staining.

## Results

### Transcriptome sequencing and assembly

To investigate the apomictic/sexual pattern expression in *E*. *curvula*, total RNA was obtained from inflorescences including all the developmental reproductive stages from two genotypes with contrasting reproductive modes: the full apomictic cultivar ‘Tanganyika’, and the sexual cultivar ‘OTA-S’. The reproductive mode of both materials was extensively checked by our workgroup through cytoembryological analysis of megagametogenesis [18]. For sequencing purposes, two biological replicates of each cDNA library were constructed from each genotype, resulting in four samples, hereafter called T3P1 and T3P2 (from Tanganyika inflorescences) and O2P1 and O2P2 (from OTA inflorescences). Pyrosequencing of the cDNA libraries conducted on a 454 GS FLX Titanium platform (Roche) generated raw reads, ranging from 339777 to 883331 reads per library; 2617197 raw reads in total (Table 2). The average length of the raw reads ranged from 358–368, with an average whole read length of ~364 bp (S1 Fig; Table 2). After filtering for adaptors, primers and low quality sequences, we obtained ~90% of high quality reads from each library, representing approximately 860 Mbp (Table 2). Raw reads were deposited in the Sequence Reads Archive (SRA) database at NCBI as BioProject 358210 “Floral transcriptome of sexual and apomictic *Eragrostis curvula*” including Biosamples SAMN06167423 (reads from OTA-S) and SAMN06167424 (reads from Tanganyika). This Transcriptome Shotgun Assembly project has been deposited at DDBJ/EMBL/GenBank under the accession GFVM00000000. The version described in this paper is the first version, GFVM01000000.

**Table 2:** Description of the raw and filtered reads for the four obtained libraries.

Input information	Raw data				Filtered data			
	O2P1	O2P2	T3P1	T3P2	O2P1	O2P2	T3P1	T3P2
# Reads	546016	848073	339777	883331	493437	771998	312337	798175
Total bases	195845384	308954685	122719547	325173669	174722770	278038711	111595560	290341744
**Length distribution**								
Mean length (bp)	358.68 ± 122.12	364.30 ± 120.18	361.18 ± 130.48	368.12 ± 128.54	354.09 ± 121.48	360.15 ± 119.63	357.29 ± 130.16	363.76 ± 128.09
Minimum length (bp)	20	18	19	19	20	18	19	19
Maximum length (bp)	1181	1183	1118	1185	1181	1183	1118	1184
Mode length (bp)	468	468	470	471	468	468	470	471
	(2784 seq)	(4521 seq)	(1962 seq)	(5384 seq)	(2570 seq)	(4167 seq)	(1832 seq)	(5016 seq)

**Fig 1:**
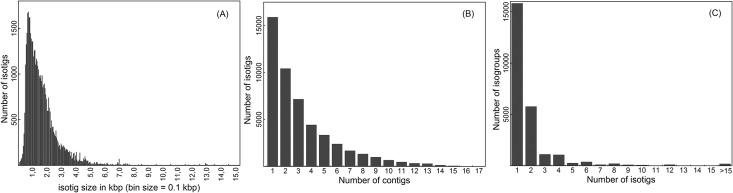
Graphic summary of the *de novo-*assembled *E*. *curvula* transcriptome. (A) Length distribution of the assembled isotigs; (B) Number of contigs used to assemble individual isotigs; (C) Number of isotigs used to assemble individual isogroups.

Due to the lack of an *E*. *curvula* whole-genome sequence, we conducted a *de novo* assembly of the high quality clean reads into transcripts. Using Newbler assembler software (v 2.6; Roche, IN, USA), 2373847 reads were *de novo*-assembled into 63763 contigs. Overlapping contigs were further assembled into 49568 isotigs (Table 3), the bioinformatic equivalent to unique RNA transcripts. Isotig length ranged from 61–15244 bp, with an overall average length of 1633 ± 1180 bp. The mode was 742 bp, with 54 sequences (Fig 1a). While 15891 isotigs were derived from single contigs, the average number of contigs per isotig was 3.2, with the highest consisting of 17 contigs (Fig 1b). More than 65% of isotigs were longer than 1000 bp, whereas ~0.2% were longer than 10 Kb. In addition, Newbler grouped isotigs originated from the same contig-graph into 25186 isogroups (equivalent to genomic locus), potentially reflecting multiple splice variants. Of those, 15808 isogroups contained a single isotig, and 5761 contained two isotigs (Fig 1c). The average number of isotigs per isogroup was 2.0, with the highest consisting of 96 isotigs.

**Table 3:** Summary of the *E*. *curvula* transcriptome assembly.

Number of contigs/isotigs	49568
Total size of isotigs	79463663
Longest isotig	15244
Shortest isotig	61
Number of isotigs > 1000 nt	32054 (64.7%; 67284024 nt)
Number of isotigs > 10 000 nt	81 (0.2%; 1039579 nt)
Mean isotig size	1603
Median isotig size	1308
N50 isotig length	1959
L50 isotig count	12 894
Isotig %A	23.51
Isotig %C	26.49
Isotig %G	26.13
Isotig %T	23.88
Isotig %N	0.00
Isotig % non-ACGTN	0.00
Number of singletons	133782
Total size of singletons	40643617
Shortest singleton	50
Longest singleton	959
**Total bp assembled transcripts**	120107280

### Assembly validation

First, assembled sequences were compared to 12995 *E*. *curvula* ESTs (GenBank EH183417 to EH195711). These were generated from four cDNA libraries (Ec01, Ec02, Ec03 and Ec04), prepared from panicles or leaves of near-isogenic lines with different ploidy levels and reproductive modes [30, 31]. Ec02 and Ec03 libraries were constructed from panicles and leaves, respectively, of the tetraploid apomictic genotype Tanganyika, and Ec01 and Ec04, from panicles of diploid and tetraploid sexual plants, respectively. BLASTN analysis revealed that 11762 (24%) of the assembled isotigs matched to 7458 (57%) *E*. *curvula* ESTs at an e-value < e^-10^, whereas at an e-value < e^-100^, 9811 isotigs (20%) matched to 6514 ESTs (50%). Thus, alignments were reanalyzed under the hypothesis that the percentages of ESTs per library might be influenced by the nature of the library considered. However, the percentages of sequences per library aligning to isotigs were similar for all libraries, although slightly greater for Ec01, followed by Ec03, Ec02 and Ec04. The fact that several isotigs did not match to ESTs and *viceversa*, suggests that both sets of sequences (i.e., ESTs plus isotigs), should be taken into account in future transcriptome-based studies of *E*. *curvula*.

We further analyzed the assembly quality by comparison with the only currently available genome of the *Eragrostis* genus, the *E*. *tef* whole genome shotgun (WGS) project (LAPY01000001 to LAPY01013883) [37]. This database consisted of 607317615 bp assembled into 13883 contigs and 4541 cDNA sequences. *E*. *tef* transcriptome sequences were aligned to *E*. *curvula* reference transcriptome, revealing that 2925 cDNAs from *E*. *tef* matched to 1836 *E*. *curvula* transcripts. The opposite analysis was conducted using *E*. *tef* cDNAs as database and querying *E*. *curvula* reference trancriptome, leading to 5913 isotigs that matched to 1708 *E*. *tef* cDNAs. Since alignments were restricted to the best match for each query to make the output manageable, differences between both sights may be explained by the miscount of some matches, particularly, the alternatively spliced forms. Taking all together, ~12% of *E*. *curvula* isotigs matched to ~65% of the 4541 available *E*. *tef* cDNAs. Despite the fact that they were obtained by searching against a small database, the low percentage of *E*. *curvula* isotigs that showed identity to *E*. *tef* encourage us to inquire about the similarity between both species. The query of our reference transcriptome against *E*. *tef* genome revealed that 65% of the contigs/isotigs matched to the aforementioned database. A subsequent analysis conducted through the software GMAP [38] showed that 60% of the reads from the four libraries of *E*. *curvula* could be mapped to *E*. *tef* genome.

As a final validation strategy for the assembly, a subgroup of isotigs was searched for the presence of chimeric assemblies. Thus, 500 sequences of at least 3850 bp with only one sequence per isogroup were selected. The sampled isotigs represented ~1% of the total isotigs and covered ~2% of the isogroups of the assembly. Expressed in bp, 2538512 out of 79463663 assembled bp (~3%) were analyzed, and, in terms of isogroups, since these sequences are representative of 2930 isotigs, they can be extrapolated to 13689335 bp of the total assembly (~17%). The analysis of the BLASTX against NCBI nr database revealed that seven of the analyzed sequences (1.4%) were chimeric, consisting of two differentiable regions aligning with different proteins (S1 Table). We then analyzed the chimeric composition of the isogroups from which the chimeric transcripts were chosen. We found that not all the sequences of each isogroup were chimeric (S1 Table). The sample was taken searching for the worse scenario, since chimeric assemblies most probably occur in larger sequences. In fact, we next took a random sample of 100 sequences ranging from 1000 to 3800 bp and none of them resulted chimeric.

### Functional annotation and classification of the *E*. *curvula* transcriptome

To annotate the *E*. *curvula* transcriptome, all assembled transcripts were compared against the nr protein database using BLASTX (*E*-value cutoff of 1e^-5^), revealing that 44506 (90%) have sequence homology to protein sequences (S2 Table). The distribution of *E*-values based on sequence homology revealed that 84.0% (37378) of the transcripts had high homology at e-values lower than 1e^−50^ and 98.4% (43808) at e-values lower than 1e^-10^. Similarity distribution showed that most transcripts (94.4%; 42046) with homologous sequences were more than 50% similar, with 5.6% with homologous sequences being 20–50% similar. Non-annotated isotigs were further compared to the NCBI EST database (dbEST) and the PROSITE database of protein domains, families and functional sites. Of the 5062 non-annotated isotigs, 2594 (5.2%) showed identity to sequences from the EST database, and 4605 (9.3%) had recognizable protein domains in the PROSITE database.

A total of 295 isotigs (0.6%) aligned with mitochondrial and/or chloroplast genomes (*E*-value < e^-50^ and coverage > 50%), or with mitochondrial and/or chloroplast proteins (*E*-value > e^-20^ and coverage > 50%).

### Classification by gene ontology (GO) annotation

**Fig 2:**
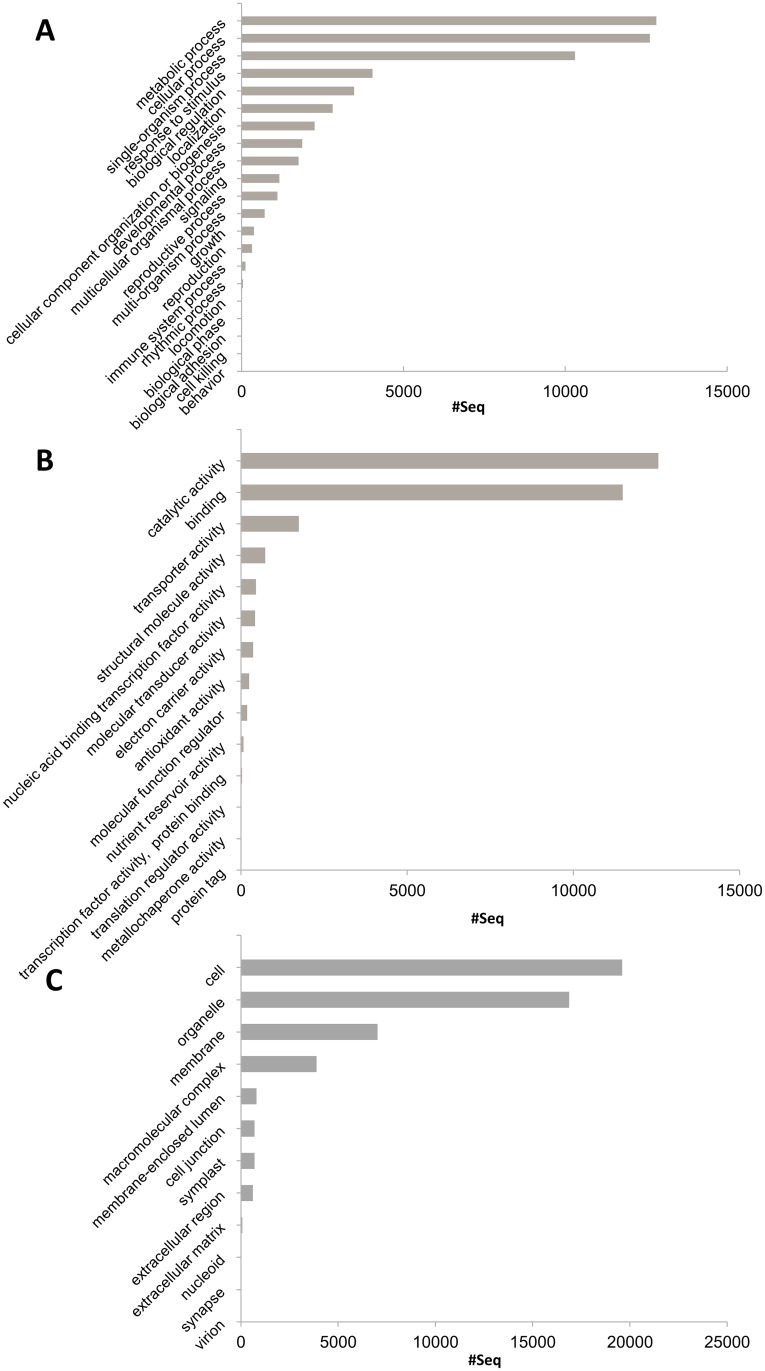
Classification of *E*. *curvula* annotated isotig sequences based on predicted gene ontology (GO) terms. Terms were obtained with BLAST2GO at level 2. (A) Biological process; (B) molecular function; (C) cellular component.

Among the 49568 isotigs identified in *E*. *curvula*, 27448 were categorized into at least one of the three main GO categories (molecular process, biological function and cellular component), which could be further subdivided into 3698 subcategories (Fig 2). Among the categorized isotigs, 7617 (27.8%) were classified into only one category, and 4333 (15.8%) into two categories. Most isotigs had more than two GO classifications, amounting to 99556 GO terms. Of these, 42440 GO terms (42.6%) represented components in the biological process category, subdivided into metabolic process (24%), cellular process (23%), and single-organism process (19%). This suggests that the inflorescences used to construct our libraries were extensively metabolically active. The cellular components category included 35121 GO terms (35.3%), with the most abundant being membrane (14%), organelle (33%) and cell (38.6%). Finally, 34864 GO terms were categorized as molecular functions (35.1%), with binding (45%) and catalytic activity (40%) being the largest subcategories.

### SSR identification in the *E*. *curvula* reference transcriptome

**Fig 3:**
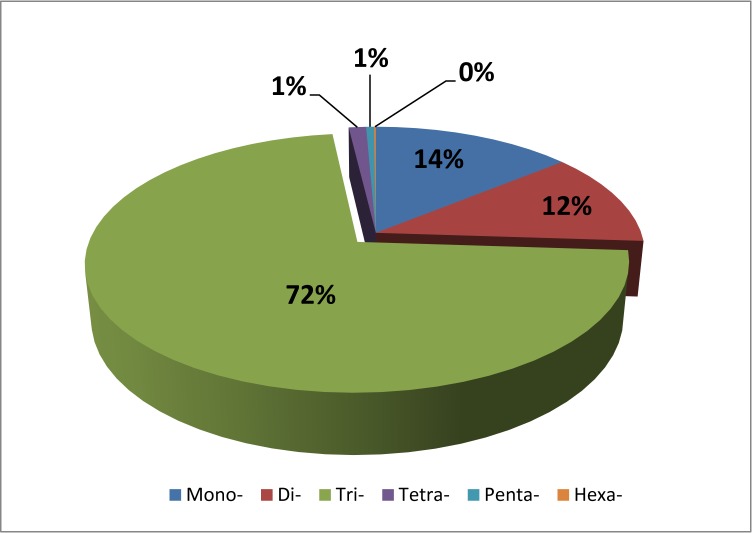
Classification of simple sequence repeats (SSRs) according to repeat unit size. The graph illustrates the frequency of different sized SSR units classified as mononucleotides (Mono-), dinucleotides (Di-), trinucleotides (Tri-), tetranucleotides (Tetra-), pentanucleotides (Penta-) and hexanucleotides (Hexa-) across the assembled *E*. *curvula* reference transcriptome.

The assembled isotigs were used as the input data sequence to develop SSRs using SSR locator software [44]. Thus, a set of 11475 SSRs was identified in 9174 assembled transcripts (Table 4), meaning that 18.5% of transcripts contained at least one microsatellite. Considering the total number of base pairs in the assembly, the SSR frequency was calculated to be 1.44 SSRs every 10 kb (Table 3), with 119 different SSR motifs (S3 Table). Considering the size of the repeat units, the most frequent motifs were the trinucleotides (72%), followed by mononucleotides and dinucleotides (14% and 12%, respectively) (Fig 3). The number of repetitions for each motif type is shown in S4 Table.

**Table 4:** Characterization of the isotig-based simple sequence repeats (SSRs) discovered using SSR locator software.

Classification	Number
Number of sequences examined	49568
Total size of examined sequences (bp)	79463663
Total number of identified SSRs	11475
Number of SSRs containing sequences	9174
Average number of SSRs per 10 kb	1.44

**Fig 4:**
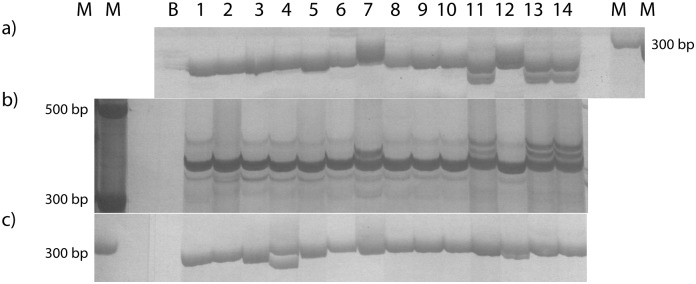
A representative profile obtained with simple sequence repeat (SSR) markers designed based on the assembled isotigs in different *E*. *curvula* cultivars. Acrylamide gel showing amplification products obtained in the 14 assayed genotypes using the primers designed based on: a) isotig06745; b) isotig28255; c) isotig 35118. References: M (Marker), B (Negative).

Specific primers were then designed based on the SSR-containing isotigs. Of these, a subgroup of 35 was synthesized, each of which was specific to a single isotig, or—eventually—to a group of isotigs belonging to the same isogroup. SSR-based specific primers were assayed in 14 *E*. *curvula* genotypes, including different combinations of ploidy and/or reproductive modes (Table 1). Amplification products were retrieved for 29 of the 35 primers (83%) assayed (Table 5 and Fig 4) whereas the remaining 17% of the primers showed no amplification on any annealing temperature tested.

**Table 5:** Gene-based SSR markers development.

Isotig name	Primer Forward	Primer Reverse	SSR Motif	Amplicon size (bp)		Polymorphisms (N° of alleles)
				Predicted	Obtained	
isotig20164	GAACTACCTGCAGATCAAGG	TGCACTTTGTTGTCATCTTC	(GAT)7	193	≈200	7
isotig38544	AAAAAGCGGAAGCAGAAC	CGCTTGAATCAGCATAGG	(GGC)7	235	*	-
isotig18488	ACAGAAACCAACAGTGGAAC	GATGGTTCCGTAGATCGTAG	(CA)6	237	≈260	4
isotig42614	GATTCAACCGTACTTCAGGA	AAAAACCTCATCTCCCCC	(AG)7	240	≈1000	2
isotig01946	TCCATATCCATCTACGAACC	GTTCGTTGTGAATTTTTGCT	(TCT)5	167	≈200	2
isotig33732	ATCCTGTTTTTGTGCTTGTT	GGGAAAAAGCTCCAGTAATC	(CCT)7	248	≈150	2
isotig35144	ACAAGTCATCGGACTCCTC	AGGGAGAGAGACGAGAAACT	(TCC)5	240	≈300	3
isotig09593	TGACATGTGACAGACTCCAT	CGTATATTGGCATCTCGTCT	(T)108	212	*	-
isotig25156	ACCACAGTACGGACACTCTC	CGTACTCCTTGTAACGCTG	(GAG)6	234	≈800	3
					≈250	2
isotig25372	GATGACAACCACAATAAGCC	ATTTCTTCCACGACGAAGTT	(CCG)5	163	≈160	5
isotig29328	AGCATCATCACCAATTCCT	GTAACGTAGGGTGGGAATC	(GCA)5	250	*	-
isotig30246	TCTTCTTCAGCCCCTTG	ACGTCGTCGTCATCCTC	(GTC)5	249	≈160	6
isotig33661	ATGGCATCTTCTCCTGTGAT	GAACCCCTTCGCAGC	(CGC)5	152	≈150	6
isotig33734	GTCCATCCGATCCCAG	AACTCATCTTTGCACGACTT	(CGC)5	225	≈250	3
isotig34134	TACTCATCGTCGTACCTCCT	GGCTGAGTACTTGGTCTCTC	(GCG)5	168	≈170	5
isotig35118	CATCATCATCCCCATTTATC	TGATCACAAGATGACCAAGA	(GAA)5	213	≈300	3
isotig07764	CCTGCTTCTTGCTCCC	AAAGAAGACGTCGTAAGCAG	(CCG)6	186	≈170	3
isotig13699	GTTCAGCATGAGTAACCACA	CTCCTTCCTCTCCTCATTCT	(AGAAGG)6	209	≈300	5
isotig18079	GACAGGACCCCTCTTCC	AGGACTCCCAGCTCAGAT	(GGC)5	155	≈150	5
isotig20053	CCACCAACCAATTATCCTAA	GATCTCGCGACAACCC	(CAC)7	233	≈250	Highly polymorphic
isotig06745	GTGAAGGAGGAGAAGTCGAG	GGAGAGCGAGTCGTCC	(CCG)5	153–150–291	≈300	4
isotig13776	TCCAACTCATCAACCAGTAA	GTAGCTCTTGCCGAACC	(CCT)5	226–227	≈230	3
isotig10159	TAGCCAGATGACCTCCAC	CCTGCTCTCCTCCGAC	(GGA)8	244–250–226	≈310	3
					≈170	4
isotig13665	TGCGCTGGACCTCTACTA	ACTGCTTCAGCCTCATCTT	(CGG)6	176–188	≈300	4
					≈200	
isotig28255	GTCGTTGAACATGATCCCT	GAGCAGGAGAACATCAAGAG	(CGC)5	177–183	≈300	5
					≈200	
isotig13242	GCTCGACTCCGTCTCC	GAAACCGTCCAAGAAGAAG	(CTT)5	184–187	*	-
isotig15838	AGTGGAGGAAGATGTAGCC	ACAGAGTTGAAGGAGCAGAG	(C)10	250–239	≈250	4
isotig25706	GGTGAGGAAGTCGAGGAT	ACTTCAAGGTGGCGTACA	(GTT)8	245–248	Unspecific	-
isotig27380	GTTCGGCGTCTCCATC	GTATATGGGCCAGTGGTTC	(TCGCCG)5	193–175	≈200	3
isotig27968	ACTTGCAGAAATCACAAAGG	GGATCGTGTTGATTGAAGTT	(A)12	160–151	≈160	Highly polymorphic
isotig28834	GCGTAGCTCTTCTCCATGA	CACTTCTTCGGGGTGTTC	(CGG)5	165–158	≈300	5
					≈160	2
isotig32396	CGCCATGAAGCATCTC	CAACTTTGTTTTTCCTCGTC	(GCC)5	229–247	≈760	2
isotig23517	CTGCATCCAGTGGTTTTC	ATGTACTCAAGGCACGACA	(GCC)6	171–165	≈160	3
isotig23773	ACCGCATTCTTTGATTTAGA	TGTGATTTCTTCGGTTCTTT	(GAG)5	210–207	≈200	7
isotig24162	TCATCTTCCTCTTGATCTGC	ATGGAAGCAGAGCACTACC	(TCG)5	248	*	-

*: No amplification

Profiles for the genotypes OTA-S (4X, sexual) and PI219928 (2X, sexual) showed greater abundance of bands, followed by Tanganyika USDA (4X, apomictic). Thus, amplifying gene SSR markers based on the assembled transcriptome allowed us to detect 108 alleles among the 14 assayed genotypes (Table 5), with the number of alleles per locus ranging from 2 to 7 (Table 5). The results obtained using SSR primers revealed a high level of polymorphism in *E*. *curvula* germplasm.

Most primers showed PCR amplicons of the expected size whereas six had larger products than expected, and two smaller (Fig 4). Three other primers displayed additional larger amplicons. All of these were polymorphic for at least one genotype (Fig 4). One primer pair gave no consistent electrophoretic patterns and accordingly was classified as highly unspecific and excluded from the global analysis.

### Differential representation of transcripts according to reproductive mode

Using a digital gene expression approach, the differential gene expression analysis according to the reproductive mode was evaluated. The use of the sexual libraries O2P1 and O2P2 as biological replicates, as well as the apomictic libraries T3P1 and T3P2, was validated prior to the conduction of the differentially expression analysis. The edgeR software provided the statistics to each assembled transcript concerning the read count contribution from individual libraries. When the contribution of reads from individual libraries, i.e., O2P1 vs O2P2 and T3P1 vs T3P2, was analyzed for each assembled transcript, the obtained correlation coefficients were 0.97 and 0.91, respectively, whereas a correlation coefficient of 0.60 was calculated when compared OP vs TP, thus validating the use of O2P1/ O2P2 and T3P1/T3P2 as biological replicates. Moreover, statistics revealed that 99.5% of the assembled contigs/isotigs were equally expressed in sexual libraries (O2P1 vs O2P2) whereas 99.2% were equally expressed in apomictic ones (T3P1 vs T3P2) (logFC > 1; p-value < 0.01). Both approaches validated the subsequent analysis of the differential expression of transcripts according to the reproductive mode. Then, we analyzed the EdgeR output statistics concerning the genotype of origin of the reads that assembled in each transcript, i.e., the individual read count from sexual or apomictic libraries (OP vs. TP) that composed each transcript, after normalization procedures (S5 Table). Thus, there were in silico identified a set of 6203 transcripts overexpressed in the apomictic libraries and 3060 overexpressed transcripts in sexual ones. It is interesting to remark that the fact that a particular transcript resulted more expressed in sexual than apomictic libraries may be explained either by its upregulation in the sexual genotypes or its downregulation in the apomictic ones. Similarly, when a particular transcript is more expressed in apomictic than sexual libraries may be due either by its upregulation in the apomictic genotypes or its downregulation in the sexual ones.

**Fig 5:**
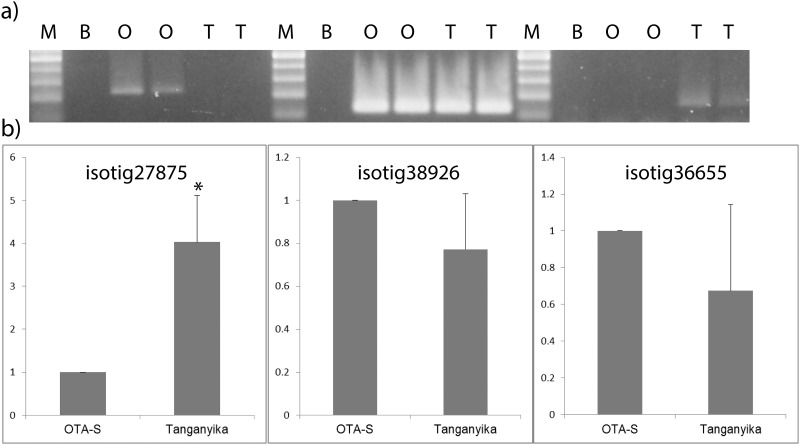
Analysis of gene expression. Reactions were performed with cDNA from inflorescences from the genotypes Tanganyika (T) and OTA-S (O). a) RT-PCR. The primer pair used are shown over the agarose gel image. Ubiquitin conjugating enzyme transcript (UBICE) was used as housekeeping gene; molecular mass markers are shown on the left; b) qRT-PCR. The primer pairs used are shown over the figures. *—significant differences between O and T for the primer pair tested (p < 0.05).

A subset of 6 transcripts were assayed with specific primers by RT-PCR and qRT-PCR, using mRNA extracted from OTA-S and Tanganyika inflorescences for validation purposes. Among the large list of differentially expressed transcripts, we first searched for those previously validated in genomic DNA using SSR-based primers. Thus, the expression of the transcripts for isotig01946, and isotig35144, *in silico* differentially expressed in Tanganyika, and isotig42614 *in silico* differentially expressed in OTA-S was tested. Additionally, newly designed primers based on isotig27875 and isotig38926, *in silico* differentially expressed in Tanganyika, were also tested (S5 Table), as well as isotig36655, equally expressed between genotypes. The primer pairs assayed retrieved amplification products, excluding those for isotig35144. The differential expression in Tanganyika and OTA-S of transcripts for isotig01946 and isotig42614, respectively, was validated through RT-PCR, since they showed a presence/absence profile (Fig 5a). The other primer combinations amplified from cDNA of both genotypes and thus were evaluated though qRT-PCR (Fig 5b). Tanganyika showed higher expression of transcripts for isotig27875 than OTA-S, whereas isotig36655, chosen as negative control of differential expression, and isotig38926, were equally expressed in both genotypes (Fig 5b). Thus, 4 out of the 5 primers that retrieved amplification products were validated

The large number of sequences identified complicates their individual analysis, therefore we sought to describe the general profiles of each set of transcripts. First, differentially expressed isotigs were subjected to deep gene ontology analysis. Out of the 3698 GOs subcategories aforementioned for the assembly, 1987 were associated to the *in silico* differentially expressed isotigs. Two sets of isotigs were generated, sexual and apomictic, and the GO annotation(s) for isotigs from each group were summarized. Near ~55% of the differential isotigs could be associated with GO terms. We found that 3546 differential isotigs in TP had 1908 GO terms and 1781 differential isotigs in OP had 1139 GO terms. The GO terms were further separated into 3 groups: GOs in common both TP and OP library, specific TP GOs and specific OP GOs, and their frequency was summarized. Isotigs differentially represented in TP and OP had 915 GOs terms in common and thus we searched for GO subcategories enriched in the apomictic or the sexual libraries. The regression analysis between the frequency of each one of the 915 GOs in both libraries revealed high correlation (R^2^ = 0.96; S2 Fig). Thus, the relative frequency of each GO in both libraries was individually tested through an ANOVA, revealing that none of the 915 GOs subcategories were differentially represented between both libraries (S2 Fig). In the two other groups, the presence of zero values for one of the sets of data precluded analysis by ANOVA. However, there were identified 961 TP unique GO terms in the second group, being found 70% in a single isotig and 20% in two different isotigs. Among the GO subcategories that were exclusively identified in the apomictic libraries, most abundant terms were considered overrepresented and notably included terms related to sexual reproduction (GO: 0019953), rRNA processing (GO: 0006364), chromatin organization (GO: 0016568), and mRNA splicing via spliceosome (GO: 0000398). In the third group, 111 OP unique terms were identified, 95% identified in a single isotig and 5% in two isotigs.

**Fig 6:**
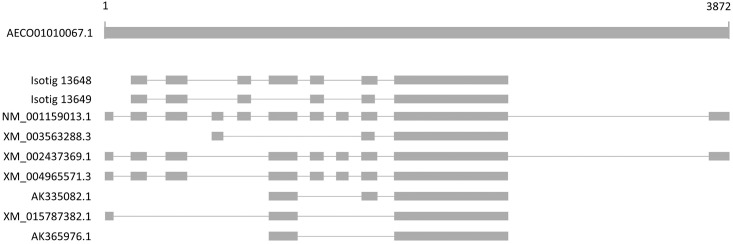
Schematic representation of an example of alternative splicing identified for the isogruop01298. Image shows the alignment among a genomic sequence from *Zea maize* (AECO01010067.1) and isotig13648, isotig13649 and seven mRNA sequences from *Z*. *maize* (NM_001159013), *Brachypodium distachyon* (XM_003563288.3), *Sorghum bicolor* (XM_002437369.1), *Hordeum vulgare* (AK365976.1), *Oryza sativa* (XM_015787382.1), *Setaria italica* (XM_004965571.3), *Triticum aestivum* (AK335082.1) proving evidence of possible alternative splicing in the *E*. *curvula* sequences under study. Numbers indicates the position in the queried sequence.

Analysis of our data also revealed noteworthy findings in terms of alternative splicing. The isotigs that we identified *in silico* as differentially represented between apomictic and sexual libraries were listed under their isogroup. Isogroups containing isotigs that were differentially identified in sexual and apomictic libraries were conserved in the list, whereas the isogroups in which isotigs were identified in one or the other library were removed. Using this strategy, there were identified 1078 isogroups with isotigs differentially represented between sexual and apomictic libraries. Since isogroups are the bioinformatic equivalent of the gene, and isotigs of the transcripts, our data revealed 1078 alternatively spliced genes in which the different forms of the transcripts are associated with the apomictic or the sexual libraries. Several isogroups were manually checked and the possible existence of alternative splicing was demonstrated. Eight out of the 45 analyzed isogroups contained isotigs corresponding to alternative spliced forms of the gene. For instance, isogroup00166 was detected as having two isotigs, isotig04248 and isotig04249, that were differentially expressed in the apomictic libraries and one isotig, isotig04247, that was identified in the sexual libraries. Interestingly, the three sequences showed similarity to three transcript variants of a *26S proteasome non-ATPase regulatory subunit 13 homolog B* from *Setaria italica*, X1 (XM_004983470.2), X2 (XM_004983471.3) and X3 (XM_004983473.3). Although the differences between *S*. *italica* and *E*. *curvula* make it difficult to speculate about the correspondence between *S*. *italica* and *E*. *curvula* variants, it is possible to demonstrate the existence of an alternative splicing process in the analyzed isogroup. The same conclusion was reached working with the isotig09588 and isotig09592, found to be differentially expressed in sexual and apomictic libraries respectively where the sequences showed identity to the putative transporter *arsB* transcript variants X1 –X6 from *S*. *italica* (XM_004985651, XM_004985653, XM_004985654, XM_004985656, XM_004985655 and XM_004985657). A different example was found analyzing the isogroup01298, with identity to variants of genes for the vacuolar sorting receptor, where isotig13648 and isotig13649 were associated to the sexual and apomictic mode of reproduction, respectively. Isotigs and public cDNAs that matched with them were compared to DNA, revealing the existence in databases of alternatively spliced forms of the gene that could be associated with the analyzed isogroup (Fig 6). In light of this initial finding, these are good candidates for future experimental validation and analysis.

## Discussion

A key goal in understanding the molecular basis of apomixis is the possible transference of this knowledge to identify traits of agricultural relevance. Achieving this goal requires a deeper comprehension of apomixis regulatory mechanisms. Since the reproductive pathways for asexual reproduction through seeds may have independently arisen multiple times during plant evolution [4], the study of most apomictic species requires the development of its own experimental model. In this sense, the existence of natural sexual and apomictic tetraploid genotypes constitutes a significant value of our strategy. For traits lacking sequence data, transcriptomic comparisons can reveal insights into the genes involved in pathway(s) of interest. Seeking to improve the understanding of the floral transcriptome of *E*. *curvula*, a reference transcriptome was *de novo* assembled using high quality reads obtained from inflorescences from two genotypes with contrasting reproductive modes: the full apomictic cultivar Tanganyika, and the sexual cultivar OTA-S. This transcriptome-based analysis strategy had been successfully applied for the study of other apomictic species [3, 45].

The lack of a standard criteria to identify misassembles or chimeric assembles makes the validation of *de novo* transcriptome assembly highly challenging, and thus several complementary strategies were conducted. First, chimerism identified in our assembly resulted lower than the predicted based on previous reports, ensuring the accuracy of the predictions based on it [46].

Then, the alignment with ESTs from the species [30] revealed a low fraction of misassembled transcripts as well as an increment in the number of available *E*. *curvula* transcripts. *E*. *curvula* transcriptome was subsequently compared to the transcriptomic and genomic sequences from *Er*. *tef* [37]. *Eragrostis* is a poorly known polyphyletic genus, with more than 400 species [47], being *E*. *tef*, an allotetraploid indigenous cereal from Ethiopia, and *E*. *curvula* complex the most studied species. The high identity values of the alignments and the level of matching sequences between *E*. *tef* and *E*. *curvula* support the involvement of *E*. *curvula* in *E*. *tef* evolution [48], and mark out that other species contributed to *E*. *tef* genome. Even when *Setaria italica* and *Sorghum bicolor* genomes have been reported as the closest to *E*. *tef* [37], the limited availability of related grasses genomes constitutes a determinant factor in the interpretation of this kind of analysis.

Finally, the high rate of SSRs correctly amplified and predicted based on isotigs constitutes indirect evidence of a high-quality transcriptome assembly. Near 83% of the isotig-based SSR primers retrieved amplification products, many more than the 58% reported in a previous study using EST-based SSRs of *E*. *curvula* [30], probably due to the meticulous manual checking of the selected primers. All the primers tested resulted polymorphic for at least one genotype, revealing a level of polymorphism in *E*. *curvula* germplasm that resulted higher than previous EST-based SSR studies [30]. There is high variability among bibliographic data in terms of the percentage of polymorphic SSRs markers developed –as low as 29% in wax gourd [49] and as high as 98% in pomegranate [50]. The deviation of the amplicons from the expected size may be due to the presence of introns [51, 52], repeat number variation, and misassembles. These factors, together with the location of the primer across splice sites [53], additionally explain the absence of amplicons. The high rate of variability observed between the materials is in agreement with previous studies with molecular markers between weeping lovegrass cultivars [54] and shows that the commercial materials used in Argentina and USA retained significant genetic diversity, which represents an excellent perspective for improvement. As they are based on functional markers, gene-based SSRs present some advantages compared with genomic DNA-based markers, mainly because changes in allelic repeats can affect protein functions. The development of more isotig-SSR-based markers that might be associated with a target trait could allow direct allele selection.

The analysis of the transcripts differentially expressed between contrasting genotypes showed reasonable correspondence between *in silico* and *in vitro* observations, suggesting that the differentially expressed transcripts reported here could drive future studies concerning differences in gene expression attributable to the reproductive mode. Moreover, it provided some useful evidences concerning GO annotation and alternative splicing. Interestingly, several GO subcategories were exclusively identified in sexual or apomictic libraries, including those related to sexual reproduction, rRNA processing, chromatin organization and mRNA splicing via spliceosome. Their participation in fating the reproductive mode in *E*. *curvula* will be subject of our following studies.

Thus, the reference transcriptome constructed based on NGS technologies constitutes a valuable database to identify gene(s) controlling key steps of the apomictic pathway in this species, and, in turn, might allow the extrapolation to other plant species. Moreover, such floral transcriptome would be useful for the identification of genes related to other agronomical traits, providing the first tools for the characterization of the biological processes involved. Additionally, it will be determinant for the conduction of array hybridization assays comparing the expression of different traits in contrasting phenotypes, such as reproductive mode and thus reducing the current number of candidates genes involved in apomixis.

## Supporting information

S1 Fig Histograms showing the length distribution of the filtered reads obtained by Roche 454 pyrosequencing of cDNA libraries from apomictic (TP1 and TP2) and sexual (OP1 and OP2) genotypes. Bin size = 12 bp.
(GIF)

S2 Fig Statistical analysis of the frequency of the GO terms identified in TP and Op libraries. a) Regression analysis of the between the frequency of the 915 GOs terms identified in TP and OP libraries; b) ANOVA analysis of the frequency of each GO term between sexual and apomictic libraries.
(DOCX)

S1 Table Analysis of the chimeric isotigs. (DOCX)

S2 Table Assembled transcripts annotation. (XLS)

S3 Table Microsatellites identified in the assembled *E*. *curvula* isotigs. (DOCX)

S4 Table Distribution of simple sequence repeats (SSRs) identified from the *E*. *curvula* reference transcriptome using SSR locator software. (DOCX)

S5 Table Statistics provided by EdgeR concerning the contribution of reads from sexual and apomictic libraries to transcript assembly. (XLSX)
