# Supplementary figures and images for: De novo transcriptome sequencing and assembly from apomictic and sexual Eragrostis curvula genotypes
Source: PLoS One. 2017 Nov 1;12(11):e0185595. doi: 10.1371/journal.pone.0185595 (PMC5665505; doi:10.1371/journal.pone.0185595)

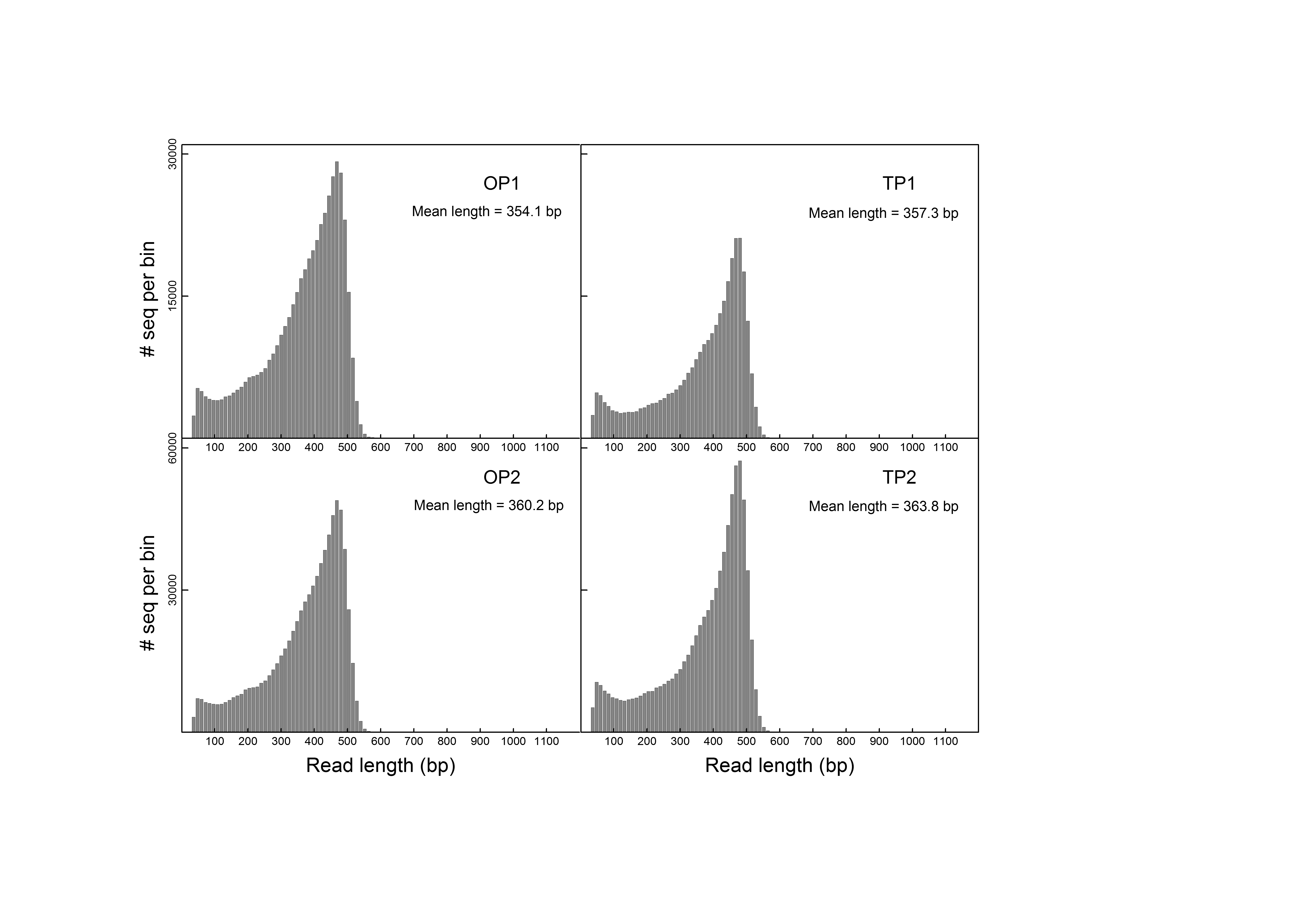

Supplement: S1 Fig — Bin size = 12 bp. (GIF) [file pone.0185595.s001.gif]
