# Supplementary material for: De novo transcriptome sequencing and assembly from apomictic and sexual Eragrostis curvula genotypes
Source: PLoS One. 2017 Nov 1;12(11):e0185595. doi: 10.1371/journal.pone.0185595 (PMC5665505; doi:10.1371/journal.pone.0185595)
Supplement: S2 Fig — a) Regression analysis of the between the frequency of the 915 GOs terms identified in TP and OP libraries; b) ANOVA analysis of the frequency of each GO term between sexual and apomictic libraries. (DOCX) [file pone.0185595.s002.docx]

S2 Fig

1. The regression was made between the 915 repeated GO terms in TP and OP libraries

|  | | | | |  |
| --- | --- | --- | --- | --- | --- |
| 1. ANOVA analysis of the frequency of each GO term between sexual and apomictic libraries.   ANOVA | | | | |  |
| Variable | N | R2 | R2 Adj | CV |  |
| Relative | 1830 | 0.0000001 | 0.000000 | 317.095267834 |  |
|  |  |  |  |  |  |
| Variance Analysis (SC tipe III) | | | | |  |
| F.V. | SC | gl | CM | F | p-value |
| Model | 0.0000000005 | 1 | 0.0000000005 | 0.000878786 | >0.99999 |
| Mode | 0.0000000005 | 1 | 0.0000000005 | 0.000878786 | >0.99999 |
| Error | 0.021953995 | 1828 | 0.000012010 |  |  |
| Total | 0.021953995 | 1829 |  |  |  |
|  |  |  |  |  |  |
| Test:LSD Fisher Alfa=0.05 DMS=0.00028 | | | |  |  |
| *Error 0.0000 gl; 1828* | |  |  |  |  |
| R. Mode | Mean | n | E.E. |  |  |
| OP | 0.001092896 | 915 | 0.000114567 | A |  |
| TP | 0.001012082 | 915 | 0.000114567 | A |  |
| Means with one letter in common are not significantly different (p > 0.05) | | | | |  |
