# Supplementary material for: De novo transcriptome sequencing and assembly from apomictic and sexual Eragrostis curvula genotypes
Source: PLoS One. 2017 Nov 1;12(11):e0185595. doi: 10.1371/journal.pone.0185595 (PMC5665505; doi:10.1371/journal.pone.0185595)
Supplement: S1 Table — (DOCX) [file pone.0185595.s003.docx]

**S1 Table. Analysis of the chimeric isotigs.**

| **Isotig*** | **Isogroup** | **Length** | **Description**** | **Isotigs/Isogroup** | **Chimeric Isotigs**  **(count)** | **Non-chimeric Isotigs**  **(count)** |
| --- | --- | --- | --- | --- | --- | --- |
| isotig04298 | isogroup00172 | 4814 | Incomplete (50%) and complete chimeras | 7 | isotig04296-isotig04298 (3) | isotig04299-04302 (4) |
| isotig11715 | isogroup00951 | 6295 | Incomplete (87% )and complete chimeras | 5 | isotig11715 (1) | isotig11716 - isotig11719 (4) |
| isotig12530 | isogroup01090 | 4663 | Complete chimeras | 6 | isotig12528 - isotig12531 (4) | isotig12532 - isotig12533 (2) |
| isotig13941 | isogroup01368 | 5805 | Complete chimeras | 6 | isotig013941 - isotig013945 (5) | isotig013946 (1) |
| isotig16256 | isogroup01893 | 6352 | Incomplete chimeras (66 and 87%) | 4 | isotig16255 - isotig16258 (4) | - |
| isotig24992 | isogroup05073 | 4898 | Incomplete (90%)and complete chimeras | 2 | isotig24992 (1) | isotig24993 (1) |
| isotig33730 | isogroup09495 | 4862 | Complete chimeras | 1 | isotig33730 (1) | - |

* The analyzed isotig is always the largest of the isogroup shown in the second column.

** Complete or incomplete chimeras, i.e., the isotigs contain the sequence of two complete or two partial proteins, or one complete and the other partial, according to BLASTX alignments. For the partial chimeras, the percentage of the protein sequences covered by the isotig is indicated between brackets.
