# Supplementary material for: De novo transcriptome sequencing and assembly from apomictic and sexual Eragrostis curvula genotypes
Source: PLoS One. 2017 Nov 1;12(11):e0185595. doi: 10.1371/journal.pone.0185595 (PMC5665505; doi:10.1371/journal.pone.0185595)
Supplement: S3 Table — (DOCX) [file pone.0185595.s005.docx]

**S3 Table. Microsatellites identified in the assembled *E. curvula* isotigs.** Microsatellites were classified according to the nucleotide composition of the repetitive motif.

| **Motif** | **Frequency** |
| --- | --- |
| (A)n/(T)n | 1603 |
| (CGC)n/(GCG)n | 1500 |
| (CCG)n/(CGG)n | 1252 |
| (GCC)n/(GGC)n | 1132 |
| (CAG)n/(CTG)n | 508 |
| (GCA)n/(TGC)n | 501 |
| (CTC)n/(GAG)n | 427 |
| (GA)n/(TC)n | 413 |
| (AGC)n/(GCT)n | 401 |
| (AG)n/(CT)n | 367 |
| (GGA)n/(TCC)n | 331 |
| (AGG)n/(CCT)n | 305 |
| (GAC)n/(GTC)n | 274 |
| (AAG)n/(CTT)n | 242 |
| (CCA)n/(TGG)n | 189 |
| (CGA)n/(TCG)n | 185 |
| (ACG)n/(CGT)n | 175 |
| (CAC)n/(GTG)n | 162 |
| (CA)n/(TG)n | 157 |
| (AGA)n/(TCT)n | 135 |
| (AC)n/(GT)n | 133 |
| (ACC)n/(GGT)n | 114 |
| (GC)n/(GC)n | 112 |
| (CG)n/(CG)n | 108 |
| (GAA)n/(TTC)n | 86 |
| (CAA)n/(TTG)n | 72 |
| (TCA)n/(TGA)n | 60 |
| (AT)n/(AT)n | 55 |
| (ATC)n/(GAT)n | 45 |
| (AAC)n/(GTT)n | 45 |
| (ACA)n/(TGT)n | 42 |
| (ATG)n/(CAT)n | 34 |
| (GATCA)n/(TGATC)n | 28 |
| (TA)n/(TA)n | 27 |
| (GTA)n/(TAC)n | 17 |
| (AGGA)n/(TCCT)n | 12 |
| (C)n/(G)n | 11 |
| (CTA)n/(TAG)n | 11 |
| (ACT)n/(AGT)n | 10 |
| (CTGC)n/(GCAG)n | 7 |
| (TCTT)n/(AAGA)n | 6 |
| (CGAC)n/(GTCG)n | 6 |
| **Motif** | **Frequency** |
| (ATAC)n/(GTAT)n | 6 |
| (TAAA)n/(TTTA)n | 6 |
| (AGCG)n/(CGCT)n | 6 |
| (AAT)n/(ATT)n | 6 |
| (ATCG)n/(CGAT)n | 6 |
| (ACGC)n/(GCGT)n | 6 |
| (TAA)n/(TTA)n | 5 |
| (GAAA)n/(TTTC)n | 5 |
| (CCTGC)n/(GCAGG)n | 4 |
| (CCGTC)n/(GACGG)n | 4 |
| (CGGG)n/(CCCG)n | 4 |
| (CATC)n/(GATG)n | 4 |
| (CAGT)n/(ACTG)n | 4 |
| (CTTC)n/(GAAG)n | 4 |
| (ATA)n/(TAT)n | 4 |
| (TTCT)n/(AGAA)n | 3 |
| (TTAT)n/(ATAA)n | 3 |
| (CCTCTT)n/(AAGAGG)n | 3 |
| (TCAC)n/(GTGA)n | 3 |
| (GGGA)n/(TCCC)n | 3 |
| (CTCG)n/(CGAG)n | 3 |
| (ATAG)n/(CTAT)n | 3 |
| (TTTTA)n/(TAAAA)n | 2 |
| (TTGA)n/(TCAA)n | 2 |
| (TGCG)n/(CGCA)n | 2 |
| (CTCCT)n/(AGGAG)n | 2 |
| (TGCTT)n/(AAGCA)n | 2 |
| (GCTC)n/(GAGC)n | 2 |
| (GCGG)n/(CCGC)n | 2 |
| (GCCG)n/(CGGC)n | 2 |
| (GCAC)n/(GTGC)n | 2 |
| (AGAGG)n/(CCTCT)n | 2 |
| (TCTTC)n/(GAAGA)n | 2 |
| (GGCTCC)n/(GGAGCC)n | 2 |
| (CCACT)n/(AGTGG)n | 2 |
| (TCACC)n/(GGTGA)n | 2 |
| (CAAC)n/(GTTG)n | 2 |
| (ATGG)n/(CCAT)n | 2 |
| (ACGG)n/(CCGT)n | 2 |
| (ACCA)n/(TGGT)n | 2 |
| (AACAG)n/(CTGTT)n | 2 |
| (AGAAGG)n/(CCTTCT)n | 2 |
| (GAACA)n/(TGTTC)n | 2 |
| (AAAG)n/(CTTT)n | 2 |
| (CTATA)n/(TATAG)n | 2 |
| (AGGGA)n/(TCCCT)n | 2 |
| (GCGA)n/(TCGC)n | 2 |
| (TGTA)n/(TACA)n | 1 |
| (TGGC)n/(GCCA)n | 1 |
| (TGCA)n/(TGCA)n | 1 |
| (TCGT)n/(ACGA)n | 1 |
| (CCTCGA)n/(TCGAGG)n | 1 |
| (TCCG)n/(CGGA)n | 1 |
| (TTCCA)n/(TGGAA)n | 1 |
| (TAAT)n/(ATTA)n | 1 |
| (TGTGG)n/(CCACA)n | 1 |
| (GTCC)n/(GGAC)n | 1 |
| (CGGGAA)n/(TTCCCG)n | 1 |
| (TCGCCG)n/(CGGCGA)n | 1 |
| (GCAA)n/(TTGC)n | 1 |
| (GAGT)n/(ACTC)n | 1 |
| (GGCTCG)n/(CGAGCC)n | 1 |
| (CTAG)n/(CTAG)n | 1 |
| (CCGAG)n/(CTCGG)n | 1 |
| (GTCCGG)n/(CCGGAC)n | 1 |
| (CCAG)n/(CTGG)n | 1 |
| (CATG)n/(CATG)n | 1 |
| (CAAA)n/(TTTG)n | 1 |
| (GATGG)n/(CCATC)n | 1 |
| (GATCC)n/(GGATC)n | 1 |
| (AGGG)n/(CCCT)n | 1 |
| (AGCT)n/(AGCT)n | 1 |
| (CACCG)n/(CGGTG)n | 1 |
| (CCACCG)n/(CGGTGG)n | 1 |
| (GCACCA)n/(TGGTGC)n | 1 |
| (ACAG)n/(CTGT)n | 1 |
| (AAAATA)n/(TATTTT)n | 1 |
