# Supplementary material for: De novo transcriptome sequencing and assembly from apomictic and sexual Eragrostis curvula genotypes
Source: PLoS One. 2017 Nov 1;12(11):e0185595. doi: 10.1371/journal.pone.0185595 (PMC5665505; doi:10.1371/journal.pone.0185595)
Supplement: S4 Table — (DOCX) [file pone.0185595.s006.docx]

**S4 Table. Distribution of simple sequence repeats (SSRs) identified from the *E. curvula* reference transcriptome using SSR locator software.**

| **Number of repetitions** | **Motif Type** | | | | | |
| --- | --- | --- | --- | --- | --- | --- |
|  | **Mononucleotides** | **Dinucleotides** | **Trinucleotides** | **Tetranucleotides** | **Pentanucleotides** | **Hexanucleotides** |
| 5 | 0 | 0 | 5372 | 121 | 26 | 10 |
| 6 | 0 | 643 | 1963 | 7 | 33 | 4 |
| 7 | 0 | 279 | 593 | 6 | 2 | 0 |
| 8 | 0 | 144 | 231 | 2 | 1 | 0 |
| 9 | 0 | 82 | 54 | 1 | 0 | 0 |
| 10 | 851 | 54 | 26 | 0 | 1 | 0 |
| 11 | 378 | 23 | 7 | 0 | 0 | 1 |
| 12 | 162 | 49 | 10 | 0 | 0 | 0 |
| 13 | 63 | 38 | 1 | 0 | 0 | 0 |
| 14 | 41 | 44 | 0 | 0 | 0 | 0 |
| 15 | 10 | 5 | 7 | 3 | 0 | 0 |
| 16 | 7 | 1 | 3 | 0 | 0 | 0 |
| 17 | 6 | 4 | 0 | 0 | 0 | 0 |
| 18 | 7 | 2 | 1 | 0 | 0 | 0 |
| 19 | 0 | 1 | 1 | 0 | 0 | 0 |
| 20 | 7 | 1 | 0 | 0 | 0 | 0 |
| 21 | 2 | 1 | 0 | 0 | 0 | 0 |
| 22 | 12 | 0 | 0 | 0 | 0 | 0 |
| 23 | 1 | 0 | 0 | 0 | 0 | 0 |
| 24 | 5 | 0 | 1 | 0 | 0 | 0 |
| 26 | 3 | 0 | 0 | 0 | 0 | 0 |
| 28 | 5 | 0 | 0 | 0 | 0 | 0 |
| 30 | 1 | 0 | 0 | 0 | 0 | 0 |
| 31 | 2 | 1 | 0 | 0 | 0 | 0 |
| 36 | 1 | 0 | 0 | 0 | 0 | 0 |
| 38 | 1 | 0 | 0 | 0 | 0 | 0 |
| 39 | 1 | 0 | 0 | 0 | 0 | 0 |
| 44 | 1 | 0 | 0 | 0 | 0 | 0 |
| >45 | 48 | 0 | 0 | 0 | 0 | 0 |
